# Consumption of snacks and energy by adolescents and overweight

**DOI:** 10.1590/1984-0462/2023/41/2022118

**Published:** 2023-03-13

**Authors:** Luiz Antonio Del Ciampo, Ieda Regina Lopes Del Ciampo

**Affiliations:** a Universidade de São Paulo, Faculdade de Medicina de Ribeirão Preto, Departamento de Puericultura e Pediatria – Ribeirão Preto, SP, Brazil.; b Universidade Federal de São Carlos, Departamento de Medicina – São Carlos, SP, Brazil.

Dear Editors of Revista Paulista de Pediatria,

The study by Lopes et al.,^1^ that evaluated associations among snacks, energy consumption, nutrients and food sources among adolescents and young adults highlights a worrying reality regarding aspects of food intake and nutritional status of individuals from the second decade of life onwards.

Studies indicate that the high consumption of snacks, usually consisting of processed foods, with excess sugar and fat and deficient in calcium and iron, is a reality in several countries,^2^ making this practice a preponderant factor for the development of chronic diseases such as obesity, diabetes, and cardiovascular disorders.^3^

In addition to nutritional aspects, physical growth, and pubertal development, which denote specific nutritional needs for this stage of life, it is also necessary to understand the emotional characteristics of adolescents that make them at high nutritional risk.^4^. Adolescents usually do not have their first meal of the day, or when they do, they avoid the consumption of milk and dairy product, and besides going several hours without eating, they often exchange traditional meals for snacks and carbonated drinks. Also, the advertising affects the adolescents’ eating habits, bringing nutritional fads, and consumption of foods of low nutritional value while in sedentary activities.^5^ As they are susceptible to influences from peer groups and the media, emotional eating predominates at this phase of life, that is, food intake frequently occurs in response to generally positive emotions.^6^

Staying away from home for many hours, whether at school or in sports/social activities, making it difficult to have meals with the family, the practicality and organoleptic attractions of snacks, the inviting atmosphere of cafeterias and the influence of peer groups exert a direct impact on the daily diet of teenagers. Another aspect that directly impacts the consumption of alternative meals is the location of fast-food stores close to schools, facilitating the access to this type of food. A study carried out by Asirvatham et al.^7^ among American adolescents showed that the high consumption of snacks was accompanied by an increase in body mass index, a direct consequence of this eating practice.

The relevance of the topic should be highlighted and serve as a warning for the implementation of outpatient follow-up services for adolescent health, with preventive actions aimed at providing guidance on good dietary practices, nutrient consumption in quality and quantity, and healthy lifestyle habits.

## References

[1] Lopes TS, Mello AV, Nogueira LR, Leme AC, Fisberg RM (2021). Energy, nutrients and food sources in snacks for adolescents and young adults. Rev Paul Pediatr.

[2] Li L, Sun N, Zhanga L, Xu G, Liu J, Hu J (2020). Fast food consumption among young adolescents aged 12-15 years in 54 low-and middle-income countries. Glob Health Action..

[3] Elisabeth L, Machado P, Zinöcker M, Baker P, Lawrence M (2020). Ultra-processed foods, and health outcomes: a narrative review. Nutrients..

[4] Gaete V (2015). Adolescent psychosocial development. Rev Chil Pediatr..

[5] Delfino LD, Tebar WR, Silva DA, Gil FC, Mota J, Christofaro DG (2020). Food advertisements on television and eating habits in adolescents: a school-based study. Rev Saude Publica..

[6] Bui C, Lin LY, Wu CY, Chiu YW, Chiu HY (2021). Association between emotional eating and frequency of unhealthy food consumption among Taiwanese adolescents. Nutrients..

[7] Asirvatham J, Thomsen MR, Nayga RM, Goudie A (2019). Do fast food restaurants surrounding schools affect childhood obesity?. Econ Human Biol..

